# Supplementary material for: Factors shaping the mental health and well-being of people experiencing persistent COVID-19 symptoms or ‘long COVID’: qualitative study
Source: BJPsych Open. 2022 Mar 21;8(2):e72. doi: 10.1192/bjo.2022.38 (PMC8987646; doi:10.1192/bjo.2022.38)
Supplement: Supplementary file 1 [file bjosup.zip › S2056472422000382sup001.docx]

**Screening questions**

**Thank you for your interest in taking part in our study on Social Distancing during Covid-19 and the impact of COVID-19 on health and wellbeing**

We need to collect some additional information to understand whether or not you are eligible to take part in the interview

**COVID-19 diagnosis status**

Positive swab test  Tested negative for antibodies

Negative swab test  Not formally diagnosed but suspected

Tested positive for antibodies  Other, please specify__________________

**If you have not received a positive test for COVID-19 or antibodies, did you experience any of the following COVID-19 Symptoms (please tick all that apply)**

A continuous cough

A high temperature

Loss or change to taste/smell

**Date that you were diagnosed or suspected that you had COVID-19**

**_ _ / _/ 2020**

**For how long after the onset of suspected/confirmed covid have you been experiencing symptoms?**

Less than 3 weeks  9-12 weeks

3-6 weeks  More than 12 weeks (please state_______________)

6-9 weeks

**Can you describe the long-term health problems that you are experiencing following confirmed or suspected COVID-19? (please tick all that apply)**

A cough  Headache

A high temperature/fever  Aches and pains

Loss or change to taste/smell  Shortness of breath

Sore throat and difficulties swallowing  Diarrhoea/vomiting

Fatigue  Sleep difficulties

Muscle pains/weakness  New onset/poor control of diabetes/hypertension

Inability to concentrate  Skin rashes

Memory lapses  Chest pains

Changes in mood, anxiety or depression  Thromboembolic conditions

Palpitations  Other, please specify__________________

**Many thanks again for your time**
